# PHAM-YOLO: A Parallel Hybrid Attention Mechanism Network for Defect Detection of Meter in Substation

**DOI:** 10.3390/s23136052

**Published:** 2023-06-30

**Authors:** Hao Dong, Mu Yuan, Shu Wang, Long Zhang, Wenxia Bao, Yong Liu, Qingyuan Hu

**Affiliations:** 1 Science Island Branch of Graduate School, University of Science and Technology of China, Hefei 230031, China; 2 Hefei Institutes of Physical Science, Chinese Academy of Sciences, Hefei 230031, China; 3 China National Tobacco Quality Supervision and Test Center, Zhengzhou 450001, China; 4 School of Electronics and Information Engineering, Anhui University, Hefei 230601, Chinabwxia@ahu.edu.cn (W.B.)

**Keywords:** defect detection, parallel hybrid attention mechanism, efficient intersection over union, spatial pyramid pooling fast

## Abstract

Accurate detection and timely treatment of component defects in substations is an important measure to ensure the safe operation of power systems. In this study, taking substation meters as an example, a dataset of common meter defects, such as a fuzzy or damaged dial on the meter and broken meter housing, is constructed from the images of manual inspection in power systems. There are several challenges involved in accurately detecting defects in substation meter images, such as the complex background, different meter sizes and large differences in the shapes of meter defects. Therefore, this paper proposes the PHAM-YOLO (Parallel Hybrid Attention Mechanism You Only Look Once) network for automatic detection of substation meter defects. In order to make the network pay attention to the key areas against the complex background of the meter defect images and the differences between different defect features, a Parallel Hybrid Attention Mechanism (PHAM) module is designed and added to the backbone of YOLOv5. PHAM integration of local and non-local correlation information can highlight these differences while remaining focused on the meter defect features. To improve the expressive ability of the feature map, a Spatial Pyramid Pooling Fast (SPPF) module is introduced, which pools the input feature map using a continuous fixed convolution kernel, fusing the feature maps of different receptive fields. Bounding box regression (BBR) is the key way to determine object positioning performance in defect detection. EIOU (Efficient Intersection over Union) is, therefore, introduced as a boundary loss function to solve the ambiguity of the CIOU (Complete Intersection Over Union) loss function, making the BBR regression more accurate. The experimental results show that the Average Precision Mean (mAP), Precision (P) and Recall (R) of the proposed PHAM-YOLO network in the dataset are 78.3%, 78.3%, and 79.9%, respectively, with mAP being improved by 2.7% compared to the original model and higher than SSD, Fast R-CNN, etc.

## 1. Introduction

The basic components in power systems are prone to physical defects due to long-term exposure to extreme weather (all-day direct sunlight, strong winds, snowstorms, storms, etc.), high mechanical tension and high pressure [1]. Traditional power system component defect detection methods mainly involve professional and experienced inspectors manually looking for defects or using detection methods to check the health of components; these approaches are time-consuming and potentially dangerous, while the detection rate is limited by the inspector’s skills [2,3]. With the new opportunities provided by intelligent power system network construction, computer vision technology is gradually being applied in the field of power systems [4]. According to the history of object detection algorithms, defect detection methods based on computer vision for power system components can be summarized into three types: image processing-based methods, machine learning methods based on manual features and deep learning methods based on convolutional neural networks.

Image processing-based defect detection methods for power system components are mainly based on features such as differences in texture or contrast between defective parts and background information for differentiation [5]. Wu et al. [6] adopted the difference between the vibration dampers and the background for segmentation using the thresholding method, used the Hessian matrix to improve the contour curvature control ability of the edge detection method, and detected the antivibration hammer in segments according to the contour features. This method has poor detection performance when the background of the antivibration hammer in the image is complex, and the use of grayscale images often causes information loss. Huang et al. [7] used local differential processing, edge intensity mapping and image fusion to complete the enhancement of antivibration hammer images, completed the segmentation of antivibration hammer by setting a threshold, and, finally, classified the degree of antivibration hammer rust according to the Rusty Area Ratio and Color Shade Index. This method can achieve good recognition results for rusty hammer in a complex background, though it is less effective in recognizing rusty hammer in low contrast. Yuan et al. [8] used IULBP (Improved Uniform Local Binary Patterns) to extract texture features from icing insulator images in the monitoring system and identify different degrees of icing insulators through the correlation coefficients of texture histograms. Since different degrees of icing insulators have obvious features, IULBP has a good recognition effect, though that effect is less satisfactory for insulators in general. Zhang et al. [9] proposed a method for the identification of conductor breaks and surface defects in transmission line UAV inspection, using adaptive threshold segmentation to extract conductor regions, detecting broken strands via square wave transform of their gray distribution curves, and, finally, identifying conductor surface defects using the projection algorithm of gray variance normalization (GVN) images of conductor regions to achieve better detection results; however, the general applicability of the method is inferior.

Traditional machine learning-based methods for detecting defects in power system components are mainly based on extracting defective features and then using classifiers, such as SVM (Support Vector Machine), to detect defective samples [10]. Dan et al. [11] advanced a method to detect glass insulators from aerial images using the Haar image feature extraction and Adaboost iterative algorithms, further segmented the insulators using color features, and, finally, analyzed the pixels obtained by segmenting the insulators to complete the insulator missing detection. However, the performance of the algorithm is not good when there is an occlusion problem in the detected image. Ullah I et al. [12] proposed a new method for defect analysis in high-voltage equipment, which first extracts rich feature maps of infrared images of the equipment using the AlexNet network, before feeding the feature maps into a random forest for training. The results show that the algorithm can effectively distinguish whether there are defects in the high-voltage equipment, though it is difficult to achieve the expected accuracy when the background of the detection image is complex. Mao et al. [13] proposed a transmission line defect identification method based on the histogram of gradients (HOG) and support vector machine (SVM) algorithms. The HOG algorithm is used for transmission line feature extraction, and the SVM algorithm determines whether a transmission line is defective based on the proposed features; its average accuracy can reach 84.3%. However, the time overhead needed by this network to detect a single image is about 539 ms, and the speed cannot meet the requirements of real-time applications. LIU et al. [14] used support vector machines to identify the state of fouled porcelain insulators in the input image in order to accurately detect the state of fouled insulators, though the recognition was not effective for images with complex backgrounds.

The deep learning-based transmission line component defect detection method mainly uses CNNs with a learning capability to extract component defect features from images autonomously, which effectively compensates for the loss of information during manual feature extraction and improves the defect detection efficiency [15]. Ni et al. [16] proposed an improved Faster R-CNN for detecting insulators, anti-vibration hammers and bird nests in transmission lines in images captured via UAVs, using the concept-ResNet-v2 network as the basic feature extraction network, and its detection accuracy was increased to 98.65%, which was achieved by fine-tuning the network parameters. However, the time overhead of this network for detecting a single image is about 676 ms, and, once again, the speed is insufficient to meet the requirements of real-time applications. JLA B et al. [17] proposed a transmission line defect detection algorithm based on an improved RetinaNet network, which uses the K-means++ algorithm to redesign the size and number of anchor frames and uses the DenseNet feature pyramid network as the backbone network. Their experimental results show that the algorithm has good real-time performance, though the accuracy is not high. Zhang et al. [18] proposed a defective insulator detection method based on the YOLO network and SPP-Net network, which used trained YOLOv5s to identify and locate insulators in the original image, before cropping them according to the locating box. The cropped image was fed into the SPP-Net classification network for defective insulator detection, and the final detection accuracy of defective insulators could reach 89%. However, connecting two networks in series increased the complexity of the model. Zhang et al. [19] proposed an improved YOLOv5-based bird nest detection method for the problem of poor applicability of transmission line bird nest detection in complex backgrounds, and the experimental results showed that the method has strong generalization capability and applicability. Bao et al. [20] proposed a BC-YOLO network based on YOLOv5 by fusing coordinate attention and the bidirectional feature pyramid network, respectively, which could accurately detect the components of transmission lines in remote sensing images, and its detection mAP reached 89.1%. Zhang et al. [21] proposed an improved YOLOv5 network for insulator detection by introducing a ghost module to reduce the model parameters and volume, while using a convolutional block attention module (CBAM) to make the network focus on the key regions of the target. Experimental results show that the improved YOLOv5 network has high detection accuracy while maintaining a low model volume.

The substation meter is an important part of the power system that can be used to monitor the use of power equipment. Substation meters are exposed outdoors for a long time, the housing easily rusts, and the dial can easily blur and crack. The method for defect detection based on computer vision has achieved certain research results in power system inspection, though in order to solve the problems of the complex background of substation meter defect images, different object sizes and large differences in appearance, this study proposes an algorithm for automatic defect detection in substation meters based on the PHAM-YOLO network. With YOLOv5 as the baseline, a PHAM module with two parallel branches is designed to pay more attention to defect features by integrating local and non-local features of meter images. The SPPF module and EIOU are introduced into YOLOv5 to adapt it to different sizes of meter and improve the accuracy of the bounding box regression of defect.

## 2. Models and Methods

YOLOv5 [22] is a one-stage object detection algorithm that is based on direct regression of the relative positions of candidate frames to achieve object localization and classification. YOLOv5 is a further improvement based on YOLOv4 [23], and the detection accuracy and speed are significantly higher. As substation meter defect images have complex backgrounds, different object sizes and large differences in shape, and they are also influenced by the shooting angle and light intensity, this paper proposes a PHAM-YOLO network model for meter defect detection on the basis of YOLOv5.

### 2.1. Architecture of the PHAM-YOLO Network

**Figure 1:**
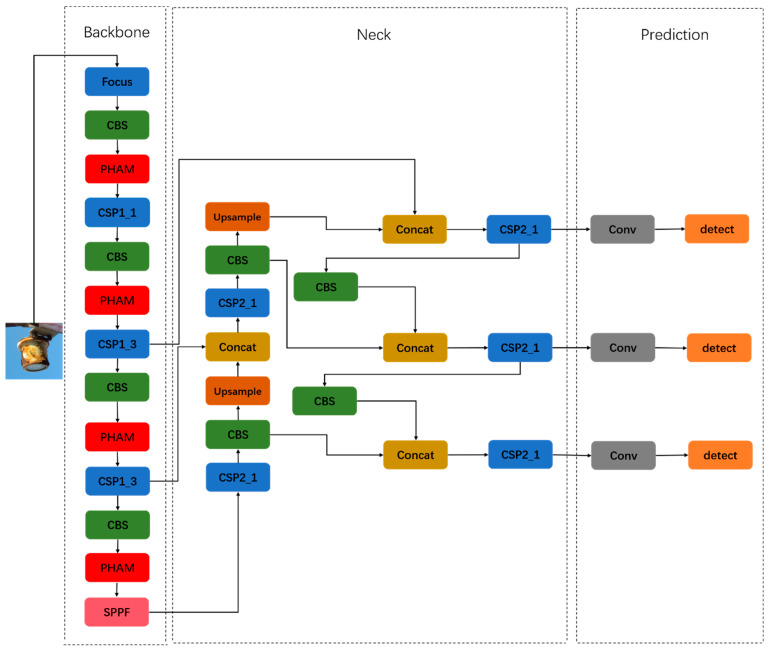
Architecture of the PHAM-YOLO network. The network consists of backbone, neck and prediction modules.

The PHAM-YOLO network model was improved on the basis of YOLOv5s, and the structure of the model is shown in Figure 1.

The model consisted of a backbone part for feature extraction, a neck part for feature fusion and a prediction part. The model used the Mosaic algorithm for data enhancement in the input part, and it stitched four random images via random deflation, random cropping and random arrangement, which effectively improved the detection of small targets. The backbone part mainly consisted of focus, CBS and CSP (Cross-Stage Partial network) structures. The focus structure performed a slicing operation; the CBS structure consisted of convolution, normalization and activation functions; and the CSP structure integrated gradient changes into the feature map to maintain higher accuracy while reducing computation. In order to make the network focus on the features of meter defects against a complex background, this paper designed and added the PHAM module to the backbone part, the inclusion of which helped the system to effectively focus on the defect features and reduced the weight input of useless background information. Spatial pyramid pooling fast (SPPF) was also used instead of spatial pyramid pooling (SPP). The SPPF structure used continuous fixed convolutional kernels to pool the input feature maps, fusing and enriching the expressiveness of the feature maps of different perceptual fields. The neck adopted the structure of FPN and PAN, where FPN used up-sampling to transmit and fuse the semantic information of different layers, while PAN effectively solved the multi-scale problem by stitching the underlying and high-level semantic information. In the prediction section, feature maps of size 80 × 80 × 255, 40 × 40 × 255 and 20 × 20 × 255 were the outputs, where 255 indicated the number of channels. The smaller the size of the feature map, the larger the image area corresponding to each cell network in the feature map and the more suitable it was for detecting large objects. The CIOU used in YOLOv5 as the boundary loss function had some ambiguity, which made the BBR regression inaccurate. Therefore, we introduced the EIOU (efficient intersection over union) loss function to solve the problems of the CIOU loss function and make the BBR regression more accurate. The remaining part of this section will describe in detail the implementation process used for the improvements and method.

### 2.2. Parallel Hybrid Attention Mechanism

**Figure 2:**
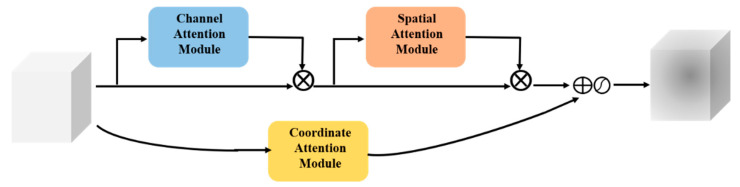
Architecture of PHAM. PHAM includes two parallel branches attention mechanisms.

**Figure 3:**
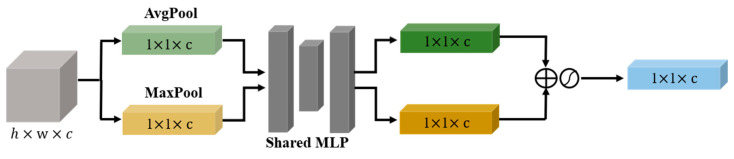
Channel attention module. The channel module utilizes a shared network to simultaneously use maximum and average pool outputs.

**Figure 4:**
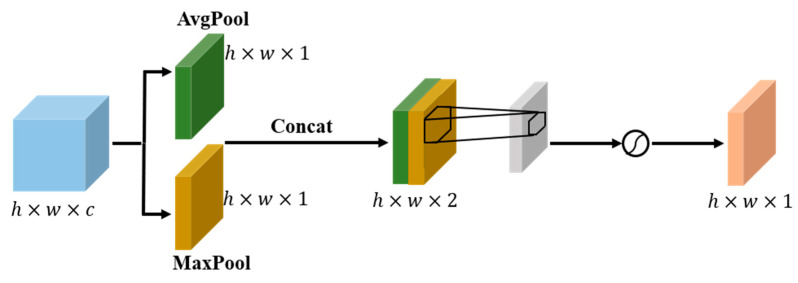
Spatial attention module. The spatial module utilizes two similar outputs that converge along the channel axis and forwards them to the convolutional layer.

**Figure 5:**
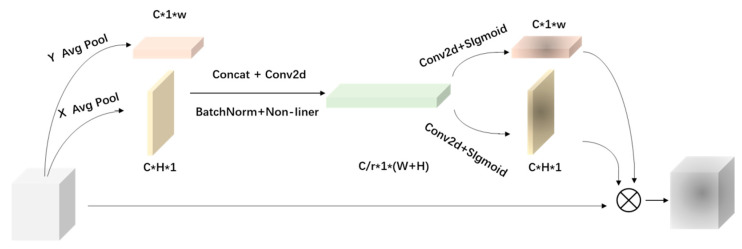
Coordinate attention module. The module decomposes channel attention into two one-dimensional feature encoding processes that aggregate features along different directions.

In order to make the network focus on the key information of the meter defects within a complex background, as well as to suppress other useless information from different channels, this paper presents a PHAM module with a two parallel branches attention mechanism, as shown in Figure 2. The upper branch was composed of channel (Figure 3) and spatial attention modules (Figure 4), and the lower branch was the coordinate attention module (Figure 5).

The channel attention of the upper branch performed a two-dimensional global pooling operation on the initial features F to obtain two sets of feature vectors, and then transmitted the two sets of feature vectors to a multilayer perceptron (MLP) network to obtain the channel attention feature map $W_{C}(F)$. Next, a 2D global pooling operation was performed on the channel level, and a convolutional kernel was used to reduce the channel dimension to 1, which could generate spatial attention feature maps $W_{s}(F)$. Next, $W_{s}(F)$ was multiplied by $W_{C}(F)$ to obtain the attention feature map output $W_{1}(F)$ using the upper branch. The channel and spatial attention of the upper branch were obtained using global pooling, capturing the local correlation of the feature map.

In the coordinate attention module of the lower branch, the input feature map was first pooled horizontally and vertically using convolution kernels of size (H, 1) and (1, W), which were averaged over the input. In order to make better use of the generated features, $Z^{h}$ and $Z^{w}$ were subjected to the concatenate operation [24], and the features after the connection were passed through the transform and the non-linear activation functions, respectively, to achieve output f:

(1) $$f=\delta (F_{1}([Z^{h},Z^{w}]))$$

where $F_{1}$ is the transform function with a convolution kernel of 1 × 1, δ is the non-linear activation function, and f is the intermediate feature mapping that encodes the spatial information in the horizontal and vertical directions. Next, f was divided into two separate tensors along the spatial dimension, and the attention feature map $W_{2}(F)$ of the output in the lower branch was obtained via convolution and non-linear processing. Therefore, the lower branch captured the non-local correlation of the feature map.

Finally, the $W_{1}(F)$ and $W_{2}(F)$ obtained from the upper branch and the lower branch were fused, and the output feature W(F) of the PHAM module were expressed as

(2) $$W(F)=\delta [W_{1}(F)+W_{2}(F)]$$

### 2.3. Spatial Pyramid Pooling Fast

**Figure 6:**
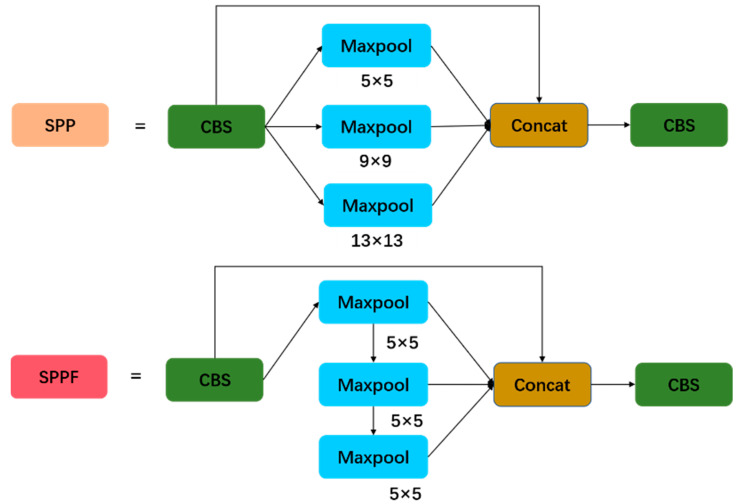
Architecture of SPP and SPPF. SPPF uses two pooling cores by default in YOLOv5, which are as follows: 5 × 5 and 1 × 1.

The SPP structure was used in the YOLOv5 network to change the size of the feature maps. As shown in Figure 6, the SPP structure first performed a CBS operation on the input feature maps, and the CBS output feature maps were then connected in cascade, with these CBS output feature maps having convolutional kernel sizes after a maximum pooling operation using 3 × 3, 5 × 5 and 9 × 9, before being fed into the CBS module. Although the SPP module implemented the function of converting the feature maps to a specific size, this parallel connection ignored the impact of different receptive field feature maps on the model performance and, with this pooling, it added additional computational overhead to the model.

The SPPF [25] structure used a continuous fixed convolution kernel to pool the input feature maps, fusing the feature maps of different receptive fields and enriching the expressiveness of the feature maps without increasing the computation. The SPPF structure replaced the parallel max-pooling operation of the three different-sized convolutional kernels in the original SPP with a serial operation of three convolutional kernels of the same size. As shown in Figure 6, the operation of the SPPF structure first performed a 5 × 5 max-pooling operation on the data transferred serially from the CBS structure. Next, the data were passed into the CBS structure via cascade splicing, which accomplished richer feature information extraction without increasing the algorithm’s computation.

### 2.4. EIOU Loss

In defect detection, the boundary loss function served to determine the positive and negative samples and evaluate the distance between the prediction frame and the true frame. The IOU (intersection over union) was the ratio of the intersection and concatenation between the prediction box and the true box, thus satisfying non-negativity, homogeneity, symmetry and triangular inequality, and had a value between 0 and 1, regardless of the size of the prediction box. In actual use, we wrote the IOU loss [26] as it is shown in Equation (4). However, the IOU loss could not optimize the case where the true and predicted boxes did not intersect, nor did it reflect the problem of how the true and predicted boxes intersected. To solve these problems, Rezatofighi et al. [27] proposed the GIOU (generalized IOU) loss, which introduced the minimum outer rectangle of the real frame and the prediction frame on the basis of the IOU, as shown in Equation (5).

(3) $$IOU=\frac{A\cap B}{A\cup B}$$

(4) $$L_{IOU}=1-IOU$$

(5) $$L_{GIOU}=1-IOU+\frac{\left|C-A\cup B\right|}{\left|C\right|}$$

where *C* is the area of the smallest outer rectangle of the prediction box and the real box. However, the GIOU loss still had some problems. The GIOU loss degenerated to IOU loss when the prediction box and the real box appeared to be contained; when the prediction box and the real box intersected, convergence was slow in the horizontal and vertical directions. Therefore, the authors of [28] proposed DIOU (distance IOU) loss and CIOU loss, which are two classes of losses that improve the speed of convergence by directly minimizing the normalized distance between the prediction frame and the true frame, and make the regression more accurate when overlapping or even containing the target frame, as shown in Equations (6) and (7).

(6) $$L_{DIOU}=1-IOU+\frac{\rho^{2}(b,b^{gt})}{c^{2}}$$

(7) $$L_{CIOU}=1-IOU+\frac{\rho^{2}(b,b^{gt})}{c^{2}}+\alpha \upsilon$$

(8) $$\alpha =\frac{\upsilon }{(1-IOU)+\upsilon }$$

(9) $$\upsilon =\frac{4}{\pi^{2}}{\left(\arctan\frac{w^{gt}}{h^{gt}}-\arctan\frac{w}{h}\right)}^{2}$$

where α is the weight function, υ is used to measure the consistency of the aspect ratio, ρ denotes the Euclidean distance between the two centroids and c denotes the diagonal length of the smallest outer rectangle between the prediction box and the real box. However, the DIOU loss did not take into account the aspect of the pre-bounded box in the regression process, and there was room for further improvement in accuracy. CIOU loss considered the width–height ratio of the regression box and the center distance between the real box and the prediction box. But, it only considered the width height ratio as the influence factor. If there were two box center points that were consistent with the original figure, the width–height ratio was the same, but the width–height value was different as, according to the CIOU loss, they may have been consistent with the regression target.

In this paper, we used the EIOU [29] loss function, instead of the CIOU loss function, as the boundary loss function, which was defined as shown in Equation (10). EIOU calculates the width–height value of the prediction box and the real box separately by separating the influence factors of aspect ratio based on CIOU. It took into account the overlapping area, the distance between centroids and the actual width–height difference. The ambiguity problems related to the CIOU loss function were solved, making the model converge faster and the BBR regression more accurate.

(10) $$L_{EIOU}=1-IOU+\frac{\rho^{2}(b,b^{gt})}{c^{2}}+\frac{\rho^{2}(w,w^{gt})}{c_{w}^{2}}+\frac{\rho^{2}(h,h^{gt})}{c_{h}^{2}}$$

### 2.5. Method Framework

**Figure 7:**
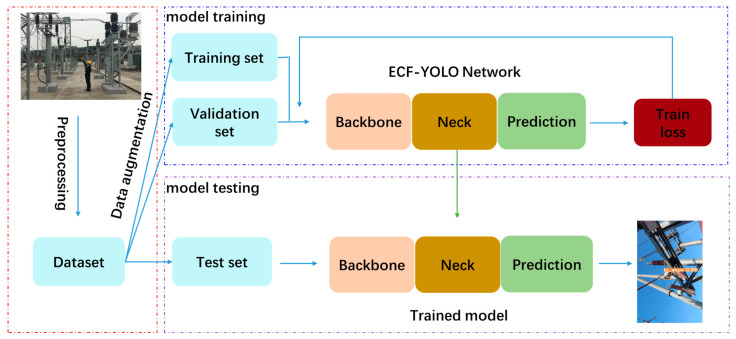
Schematic framework of our Meter Defect Type detection network.

The flow of the substation meter defect detection algorithm based on the PHAM-YOLO network and presented in this paper is shown in Figure 7.

We used the meter defect images from the manually inspected process to construct the substation meter defect dataset used in this algorithm. The images in the dataset were randomly divided into training, validation and test sets in the ratio of 6:2:2. In order to enrich the training set and improve the robustness of the model, the images in the training set were horizontally mirrored and rotated by 10°. In order to improve the accuracy of the model, this paper proposes a PHAM-YOLO network by designing a PHAM attention module, using the SPPF module instead of the SPP module, and using EIOU loss instead of CIOU loss on the basis of YOLOv5, respectively. In the training process of the PHAM-YOLO network, the original image and label file were, firstly, input into the model, and the prediction box of the image was predicted via the model; the loss value was calculated from the prediction box and the real box, which was then passed back to the model to optimize the weight parameters, and the loss value was reduced and the model performance was enhanced after several training iterations. The final trained model weights were tested on the test set to determine the test results.

## 3. Dataset

**Figure 8:**
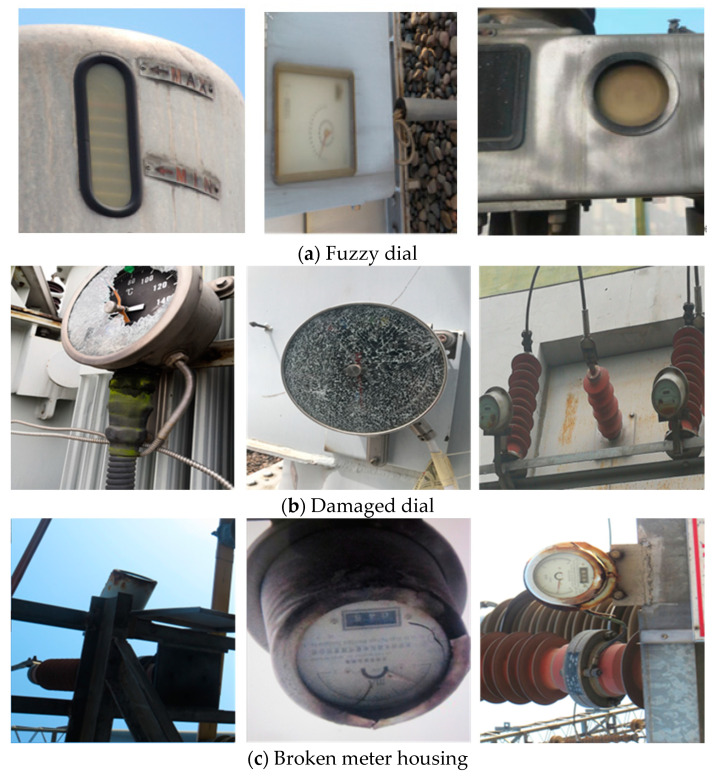
Example of meter defect.

Since there is no publicly available dataset of substation meter defect images on the network, in order to verify the performance of the algorithm in this paper, a dataset of substation meter defects was constructed from images taken via manual inspection. When selecting these images, in order to ensure the richness of the dataset, as far as possible, images with different angles, backgrounds and shooting distances were selected, aiming to ensure a balance of different target categories. The meter defect images obtained are then divided into three categories: fuzzy dial (as shown in Figure 8a), damaged dial (as shown in Figure 8b) and broken meter housing (as shown in Figure 8c).

**Figure 9:**
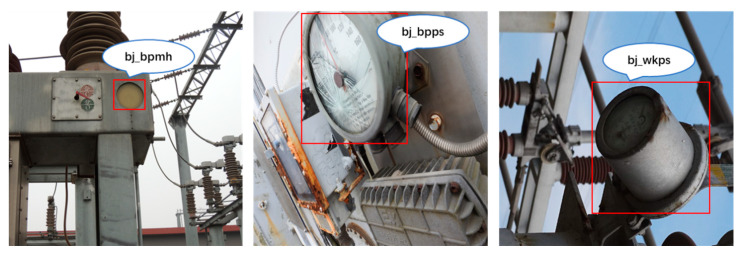
Example of data annotation.

The purpose of data annotation is to mark the position and category of the target in each image. In order to ensure the reasonableness and accuracy of the labelling, the data in this paper were manually labelled using the LabelImg labelling software under the guidance of professionals. For the substation meter defect dataset, if the meter dial was blurred, it was labelled as “bj_bpmu”; if the meter dial was broken, it was labelled as “bj_bpps”; and if the meter housing was broken, it was labelled as “bj_wkps”. Some examples of data annotation are shown in Figure 9. The annotated data are also created strictly in accordance with the public dataset PascalVOC [30] format, and the annotation file is in .xml format.

**Figure 10:**
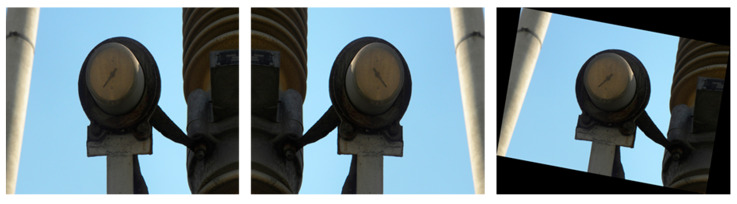
Examples of partially expanded images.

A total of 1439 images of auxiliary equipment defects were selected from the images acquired during the manual inspection, including 598 images of blurred dials, 537 images of broken dials and 304 images of broken housings. These three types of auxiliary equipment defect images were divided into training, validation and test sets in the ratio of 6:2:2. Due to the constraints on the practical situation, the equipment defect images obtained during the manual inspection are limited. On the other hand, for the deep learning model, a rich dataset is beneficial to the performance improvement of the model, and it can also improve the generalization ability of the model. Therefore, in this section, the training set images in the dataset are horizontally mirrored and rotated by 10°, which can simulate the difference of target shape and size due to different angles when shooting manually, on one hand, and enrich the training set to avoid overfitting during the network training, on the other hand. The data generated after expansion are shown in Table 1 and the visualization of its extension is shown in Figure 10.

## 4. Experimental Results and Analysis

### 4.1. Experimental Environment and Parameters

The experiment was implemented using the Ubuntu 18.04 LTS 64-bit operating system, and the graphics card model used was NVIDIA GTX 2080 Ti. Python 3.7 and the Pytorch 1.5 framework were used for training. The training parameters of the network are as follows: the image input size is 640 × 640 × 3, the initial learning rate is 0.01, the total number of training epochs is 150, the optimization algorithm is the SGD algorithm and the batch size for both training and testing is 16.

### 4.2. Performance Evaluation Metrics

For multi-category target detection models, the most commonly used performance evaluation metric is mAP (Average Precision Mean), which measures the overall detection effectiveness of the model, with larger values indicating the higher accuracy of the model in localization and recognition. The defined equation is shown in Equation (11), in which AP is the average precision of a single target class, as shown in Equation (12). P (Precision) represents the proportion of correctly predicted boxes and R (Recall) represents the proportion of all true boxes that are predicted. In this paper, we used the precision, recall and mAP as evaluation metrics to measure the effectiveness of our PHAM-YOLO network. For deep learning models, in addition to evaluation metrics related to performance aspects, there are also aspects related to model efficiency. Thus, in this paper, we mainly choose FLOPs (floating point operations) and network model size (MByte) as the evaluation metrics related to efficiency.

(11) $$mAP=\frac{\sum_{i=1}^{N}AP}{N}$$

(12) $$AP=\int_{0}^{1}P(R)d(r)$$

(13) $$P=\frac{TP}{TP+FP}$$

(14) $$R=\frac{TP}{TP+FN}$$

### 4.3. Results and Analysis

#### 4.3.1. Ablation Experiments

**Figure 11:**
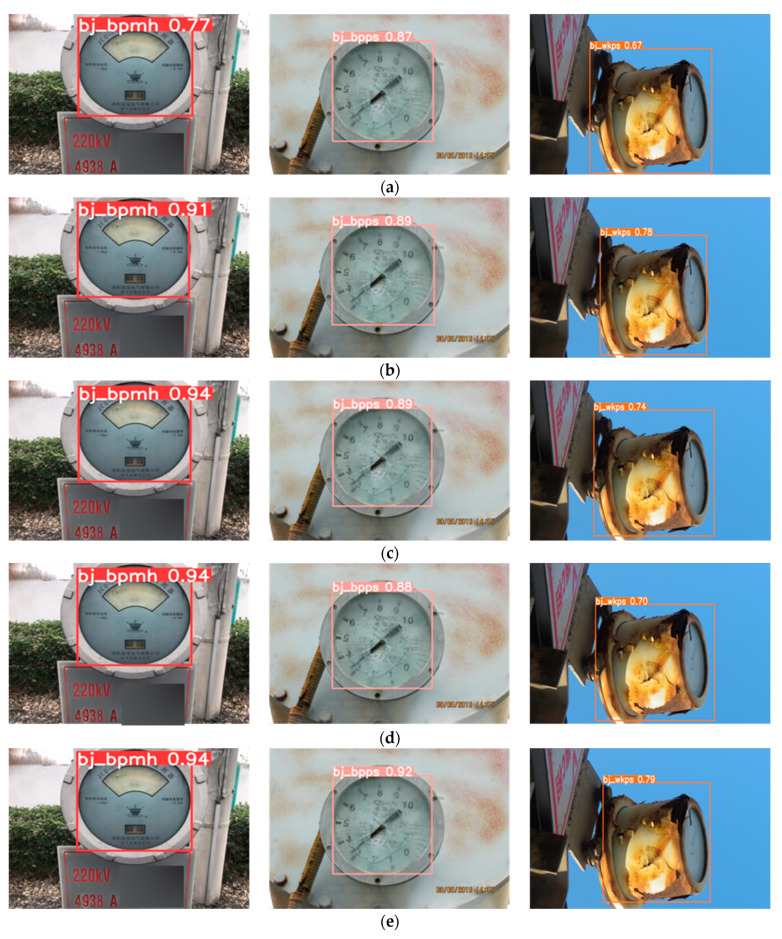
Visualization of test results generated by combining different modules with YOLOv5. (**a**) YOLOv5. (**b**) YOLOv5 and EIOU. (**c**) YOLOv5 and PHAM. (**d**) YOLOv5 and SPPF. (**e**) PHAM-YOLO.

In order to verify the impact of the proposed improvements on the model’s performance, this section uses the original YOLOv5 as the baseline network, replaces the CIOU loss function with the EIOU loss function, adds the PHAM attention mechanism and uses the SPPF module instead of the SPP module, and the experimental results are shown in Table 2. The experimental results show that the proposed improvements can improve the performance of the network for the substation meter defect dataset. The EIOU loss function solves the BBR regression inaccuracy problem and improves the mAP of the model by 1.3%. The SPPF module integrates the feature maps of the different receptive field and improves the expression ability of the feature maps, which improves the mAP of the model by 1.4%. The most excellent performance improvement is the PHAM attention mechanism, with a mAP improvement of 2.2% relative to the original network. This result shows that for complex detection tasks, such as substation meter defect detection, the attention module enables the network to focus on critical information about meter defects in complex contexts and suppress other useless information from different channels. When the three modules are fused, mAP is the highest, improving by 2.7% relative to the original network. Figure 11 shows the results of fusing different modules on the base of YOLOv5, and it is clear that the fusion of the three modules can effectively improve the localization and classification of the original YOLOv5 network and improve the performance of the modules.

#### 4.3.2. Comparison of Different Attention Modules

**Figure 12:**
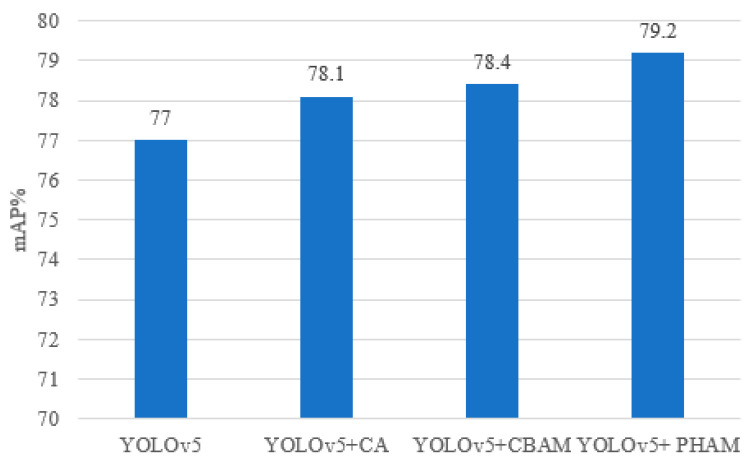
Effect of different attention modules on model performance.

The experiments compare the effects of adding different attention mechanism modules to the backbone module of YOLOv5 based on the performance of the substation meter defect detection network, and the results are shown in Figure 12. As can be seen from Figure 12, the performance of the model is improved after integrating the CA, CBAM [31] and PHAM modules. This result indicates that the attention module has a better impact in terms of improving the model’s performance in detection task with a complex background, such as in substation meter defect detection. However, the CA module is unable to capture long-distance dependencies due to the need to calculate attention weights for the entire feature map. The CBAM module adds the target location information to the feature map channel via a global pooling operation, and although it captures the local information, it always fails to obtain the long-range dependent information. Compared to the CA and CBAM modules, the PHAM module fuses local attention information and global attention information to obtain more abundant semantic information on the basis of using space and channel, which can be applied to the input feature maps in a complementary way to enhance the performance of the network. Therefore, for the substation meter defect dataset described in this chapter, the PHAM module has the best result, with a 2.2% improvement in mAP.

**Figure 13:**
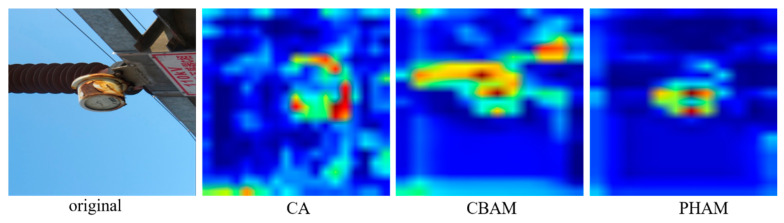
Comparison of the heat map after model fusion for each attention module.

Figure 13 gives the heat map of the test images in the comparison experiments of YOLOv5 that fuse each attention module. The color in the figure indicates the level of attention the model pays to the image, where light green or light blue indicates low attention, yellow or orange indicates average attention, and red indicates heavy attention. Figure 13 shows that, for images with complex backgrounds, such as meter defects, adding the attention mechanism to the network model allows the model to focus on the differences between the defect features and improve the performance of the model. Compared to the CA and CBAM modules, PHAM attention is focused on both channel information and location information, suppressing other useless information from different channels, and it is, therefore, also better for the judgment of the correct classification.

#### 4.3.3. Spatial Pyramid Pooling Comparison Experiment

In order to verify the superiority of the SPPF module used in this paper for substation meter defect detection, the RFB [32] (Receptive Field Block) and ASPP [33] (Atrous Spatial Pyramid Pooling) modules were used instead of the SPP module, for which the experimental results are shown in Table 3. From the table, it can be seen that the mAP of the SPP module has the lowest values, mainly due to the fact that the SPP module cannot fuse well the local and overall information of feature maps when extracting different receptive field information, resulting in the loss of some feature map information, which affects the accuracy of the model. The RFB module is a multi-branching cavity convolution module that draws on the feature law of the human visual receptive field, using different cavity convolutions to change the receptive field of the convolution layer, as well as changing the number of channels by 1 × 1 convolution to expand the receptive field of the model. Similarly, the ASPP module is similar to the RFB module, as it also expands the receptive field of the model by convolving different voids with different expansion rates. Compared to the original SPP module, the RFB and ASPP modules increase the mAP of the model at the cost of a complex computational overhead, especially the ASPP module, which increases the mAP of the model by 1.2%, as well as doubling the model size. Compared to the other three modules, SPPF improves the mAP of the model by 1.4% without additional computational overhead, and its advantage for the substation meter defect dataset is significantly higher than those of the other modules.

#### 4.3.4. Comparison of Different Object Detection Networks

To further verify the effectiveness of the PHAM-YOLO network for meter defect detection, the algorithm, in this chapter, is also compared to the current mainstream target detection algorithms, and the results of the comparison experiments are shown in Table 4. Compared to the mainstream defect detection algorithms, YOLOv5 has obvious advantages, and its mAP is higher than those of SSD, YOLOv4 and other networks. The PHAM-YOLO network model proposed in this paper proposes three improvements to substation defect images. Therefore, the PHAM-YOLO network model has the highest P, R and mAP values, which shows that the algorithm outlined in this paper can effectively improve the locating of meter defects and identification of their defect types. In summary, the PHAM-YOLO network proposed in this chapter has a good detection effect for the substation meter defect dataset in natural scenarios and can distinguish different meter defect types more precisely.

## 5. Discussion

**Figure 14:**
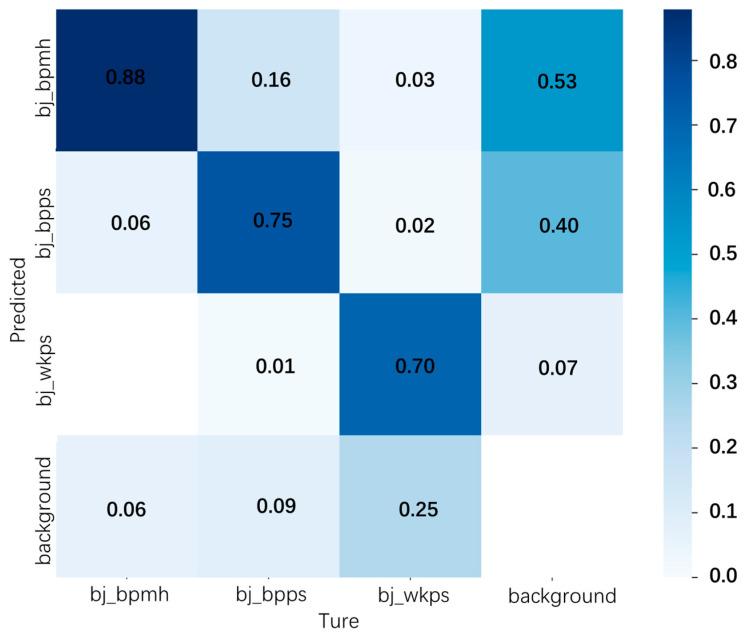
Confusion matrix for PHAM-YOLO network detection.

The confusion matrix, also known as an error matrix or likelihood matrix, is a commonly used result visualization tool in the field of deep learning, and it is mainly used to analyze detection results and errors. The confusion matrix of the detection results of the model in this paper is shown in the following Figure 14, from which we can see that there is a false detection between the fuzzy dial and broken dial. This problem is due to the similar characteristics of the two defects, as well as the fact that, sometimes, the broken dial is also blurred. The meter housing is mainly broken because the meter is exposed to the elements and rusts easily, and there are also other rusted metal materials in the substation, which can easily lead to the false detection of broken meter housing; thus, the complex background of the substation has a greater impact on the broken housing.

Although the proposed PHAM-YOLO network effectively improves the meter defect detection accuracy by designing PHAM and introducing SPPF module and EIOU loss, further research is needed to solve the problems presented during image acquisition, such as the environmental illumination conditions variation, different vision and the imbalance in defect data.

## 6. Conclusions

The meter defect images taken during manual inspection have problems, such as their complex backgrounds, different target sizes and large differences in appearance, while the three defects have similarities, making it difficult for the existing model to accurately detect and distinguish the three defects. To address the above problems, a PHAM-YOLO network model based on YOLOv5 is proposed. The main contributions are as follows:

(1) The PHAM module can focus the network on key areas in the complex background of the meter defect image and the differences in various defect features, highlighting the differences in meter defect features.
(2) The SPPF module uses continuous fixed convolution kernels to pool the input feature maps and fuses the feature maps of different receptive fields, which does not increase the computation.
(3) The EIOU loss function solves the ambiguity problem of CIOU loss and makes the BBR regression more accurate.

The experimental results show that for the substation meter defect dataset, the recognition accuracy of the PHAM-YOLO network proposed in this paper is higher than those of other mainstream target networks, which can greatly help substation staff to solve the manual inspection problem. In addition to the meter defects, some other components in power systems may also have defects; thus, the proposed method also provides some ideas for detecting other component defects in power systems.

## Figures and Tables

**Table 1:** Substation meter defect data set statistics.

Classes	Training Set	Validation Set	Test Set
bj_bpmh	1074	120	120
bj_bpps	963	108	108
bj_wkps	546	61	61
Total	2583	289	289

**Table 2:** Results of ablation experiments.

Models	EIOU	PHAM	SPPF	P (%)	R (%)	mAP (%)
YOLOv5	×	×	×	77.9	77.2	77.0
	√	×	×	75.0	79.0	78.3
	×	√	×	79.5	76.1	79.2
	×	×	√	80.3	75.8	78.4
	√	√	√	78.3	78.3	79.7

**Table 3:** Effect of different spatial pyramidal pooling on performance.

Models	mAP (%)	FLOPs (10^9^)	Size (M)
YOLOv5 and SPP	77.0	8.1	14.5
YOLOv5 and RFB	78.0	8.4	15.8
YOLOv5 and ASPP	78.2	11.3	31.0
YOLOv5 and SPPF	78.4	8.1	14.6

**Table 4:** Detection results for some mainstream object detection networks.

Models	P (%)	R (%)	mAP (%)
YOLOv3	63.9	64.5	64.3
YOLOv4	73.4	72.6	74.3
SSD	77.1	68.8	71.4
Faster R-CNN	59.9	78.9	72.8
YOLOv5	77.9	77.2	77.0
PHAM-YOLO	78.3	78.3	79.7

## Data Availability

The research data can be found at: https://github.com/wine1315923576/test.
